# Incidence of surgically treated chronic subdural hematoma after head injury with normal initial computed tomography

**DOI:** 10.1007/s00701-024-06040-y

**Published:** 2024-03-22

**Authors:** Aaro Heinonen, Minna Rauhala, Harri Isokuortti, Rahul Raj, Anneli Kataja, Milaja Nikula, Juha Öhman, Grant L. Iverson, Teemu Luoto

**Affiliations:** 1 The Faculty of Medicine and Health Technology, Tampere University, Arvo Ylpön katu 34, 33520 Tampere, Finland; 2 Department of Neurosurgery, Tampere University Hospital, Tampere, Finland; 3 Department of Neurosurgery, Helsinki University Hospital and University of Helsinki, Helsinki, Finland; 4 Department of Radiology, Medical Imaging Centre, Tampere University Hospital, Tampere, Finland; 5 Department of Physical Medicine and Rehabilitation, Harvard Medical School, Boston, MA USA; 6 Department of Physical Medicine and Rehabilitation, Spaulding Rehabilitation Hospital and the Schoen Adams Research Institute at Spaulding Rehabilitation, Charlestown, MA USA

**Keywords:** Chronic subdural hematoma, Computed tomography, CT negative, Head injury

## Abstract

**Purpose:**

The objective was to determine the incidence of surgically treated chronic subdural hematoma (cSDH) within six months after head trauma in a consecutive series of head injury patients with a normal initial computed tomography (CT).

**Methods:**

A total of 1941 adult patients with head injuries who underwent head CT within 48 h after injury and were treated at the Tampere University Hospital’s emergency department were retrospectively evaluated from medical records (median age = 59 years, IQR = 39–79 years, males = 58%, patients using antithrombotic medication = 26%). Patients with no signs of acute traumatic intracranial pathology or any type of subdural collection on initial head CT were regarded as *CT negative* (*n* = 1573, 81%).

**Results:**

Two (*n* = 2) of the 1573 *CT negative* patients received surgical treatment for cSDH. Consequently, the incidence of surgically treated cSDH after a normal initial head CT during a six-month follow-up was 0.13%. Both patients sustained mild traumatic brain injuries initially. One of the two patients was on antithrombotic medication (warfarin) at the time of trauma, hence incidence of surgically treated cSDH among patients with antithrombotic medication in *CT negative* patients (*n* = 376, 23.9%) was 0.27%. Additionally, within *CT negative* patients, one subdural hygroma was operated shortly after trauma.

**Conclusion:**

The extremely low incidence of surgically treated cSDH after a normal initial head CT, even in patients on antithrombotic medication, supports the notion that routine follow-up imaging after an initial normal head CT is not indicated to exclude the development of cSDH. Additionally, our findings support the concept of cSDH not being a purely head trauma-related disease.

## Introduction

Chronic subdural hematoma (cSDH) is a frequent neurosurgical condition [26]. The incidence of cSDH is highest in the elderly, with reported incidence rates of 46–58/100,000/year in people over 65 years of age [1, 13, 29]. The incidence has risen presumably because some of the main risk factors of cSDH, including increased age and use of antithrombotic medications, have become more prevalent [29]. Historically, the condition was considered directly trauma related [25]. Especially trauma of the bridging veins of the cerebral cortex was believed to be the origin of bleeding. More complex etiology has been proposed in recent years, although prior trauma has still been considered the main causative factor [14]. cSDH is hypothesized to form in part by an inflammatory process originating from trauma to dural border cells. Inflammatory cells attempt to repair the border cell damage—but instead cause membrane and blood vessel formation to the affected subdural region. These fragile and permeable neovessels bleed and result in fluid accumulation to subdural space. The fluid accumulation maintains the inflammatory processes and further promotes the growth of the hematoma which eventually causes the clinical signs and symptoms of cSDH [10]. Spontaneous de novo formation with no preceding trauma has been characterized as well [24]. In addition to trauma, increased age [1, 11], and the use of antithrombotic medication (including anticoagulants and antiplatelets) [2, 6, 7, 12, 30], another risk factor for cSDH is alcohol misuse [25]. Additionally, subdural hygroma, an accumulation of cerebrospinal fluid in the subdural space, has been proposed as an etiologic factor for cSDH [22, 27, 32]. Surgical evacuation of subdural hematoma via burr hole accompanied with subsequent draining remains the primary treatment option for symptomatic patients[33].

There is limited research on the development of cSDH after head traumas with normal initial computed tomography (CT) scans. The prior literature consists mainly of case reports [5, 20]. The objective of this study was to determine the incidence of surgically treated cSDH within six months from a head injury in a series of consecutive adult patients with normal initial head CT scans. Additionally, we aimed to determine if there are any identifiable pre- or peri-injury risk factors for surgically treated cSDH after head injuries with normal initial CT scans.

## Methods and materials

### Material and ethics

This study is a part of the Tampere Traumatic Head and Brain Injury Study. All consecutive patients with head injuries who were treated and CT scanned at the Tampere University Hospital’s emergency department, between August 2010 and July 2012, were retrospectively evaluated from the hospital’s patient records. A total of 3023 head injuries in 2908 patients were identified. A six-month follow-up period for the development of cSDH was included in the data collection.

**Fig. 1:**
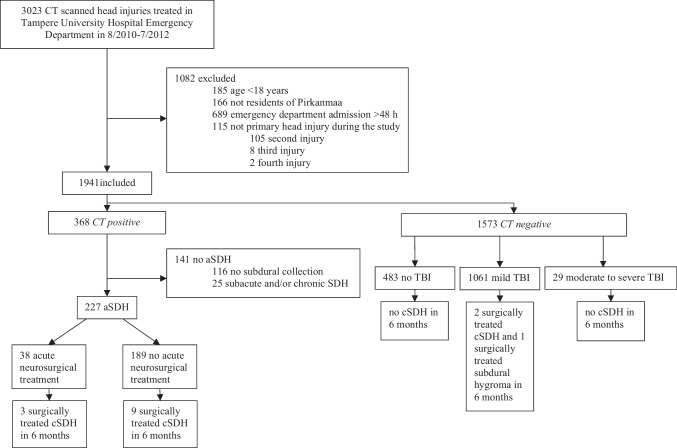
A flowchart of the study sample

This study focused on adult (18 years or older) patients who were residents of the Pirkanmaa region at the time of injury and were clinically evaluated and scanned with head CT at the Tampere University Hospital’s emergency department within 48 h (≤ 48 h) after head injury. Patients who had suffered more than one head injury during the study period were included only once in the study sample with the initial head injury as the index injury. A total of 1941 adult patients undergoing acute head CT following injury were identified. A flowchart of the study sample is provided in Fig. 1.

The Pirkanmaa region is a geographically well-defined area with both rural and urban areas that holds one of Finland’s five university hospitals with a neurosurgical service (Tampere University Hospital, Tampere, Finland). During the study period, Pirkanmaa had 490,000 residents, which comprised 9% of the total population of Finland (5.4 million) at the time. In addition to Tampere University Hospital, there is one local hospital with a CT scanner used for patients with head injuries in the Pirkanmaa region. However, most of the head trauma patients, and all the patients requiring neurosurgical care, are evaluated at the Tampere University Hospital.

The study was approved by the Ethics Committee of the Pirkanmaa Hospital District, Tampere, Finland (ethical code: R10027). All data was collected retrospectively without contacting the patients, therefore no written informed consent was obtained or required.

### Data collection

A detailed and structured data collection was performed from the patient’s medical records. Variables collected included demographics, antithrombotic medication (including anticoagulants and antiplatelets), injury-related information, clinical TBI indices, emergency head CT findings (acute traumatic lesions), acute neurosurgery due to TBI, and follow-up findings in relation to cSDH. Minimal criteria for TBI were based on the World Health Organization’s (WHO) definition [3]. Cases with a Glasgow Coma Scale (GCS) score < 13 after 30 min post-injury, post-traumatic amnesia greater than 24 h, and/or loss of consciousness more than 30 min were coded as moderate to severe TBI. There was a considerable number of patients with missing GCS scores. These patients were coded based on clinical findings and examinations reported in the patient’s medical records. Not all the patients had documented clinical signs of TBI but were likely CT scanned because of age, antithrombotic medication, or concern about the mechanism of injury based on the judgment of the on-call physician. Referral criteria for acute head CT were based on the former Scandinavian guidelines for the initial management of minimal, mild, and moderate head injuries [16]. Two neuroradiologists examined all the head CT scans. Study data was collected before the National Institute of Neurological Disorders and Stroke and Common Data Elements (CDEs) [9] for traumatic brain injury (TBI) imaging were established. However, all CDEs possible with non-contrast structural CT were included [17].

All patients were CT scanned within 48 h (≤ 48 h) after head injury and divided into two groups: *CT positive* and *CT negative*. A patient was regarded *CT positive* if there were any signs of acute traumatic intracranial pathology on initial head CT. Because the aim of this study was to examine the development of cSDH (and eventual surgical treatment) after normal initial head CT, the patients with any kind of subdural collection on initial head CT were regarded as *CT positive*. Consequently, patients with no signs of acute traumatic intracranial pathology or any type of subdural collection on initial head CT were regarded as *CT negative*. CT negative patients may have had other lesions or abnormalities on the initial head CT (e.g., lesions of vascular etiology).

The main outcome variable was defined as *surgical treatment of cSDH.* In the absence of strict universal criteria, we used the following definition for cSDH: a predominantly hypodense blood collection in the subdural space on head CT (or on magnetic resonance imaging (MRI)). No structural follow-up was conducted, and patients were CT scanned only if neurological symptoms emerged. Hence, surgical treatment of cSDH was the most reliable outcome measurement available.

### Statistical analysis

IBM SPSS Statistics for Windows (version 29, IBM Corp.) was used for data analyses. Descriptive statistics (frequency, percentage, median, interquartile range (IQR)) were used to describe variable characteristics. Blaker’s binomial confidence intervals (CI) (95% confidence level) were calculated with R (version 4.3.2).

### Data availability statement

The data that support the findings of this study are available from the corresponding author upon reasonable request.

## Results

### Characteristics of the cohort

The cohort included 1941 patients with a median age of 59 years. Of the 1941 patients, 1122 (58%) were men. The most common mechanism of injury was ground-level fall in 1016 (52%) patients. Alcohol intoxication was reported for 567 (29%) patients, and 494 (26%) patients were on antithrombotic medication at the time of injury. Most of the patients, 1269 (65%), had a mild TBI. Moderate to severe TBI was reported for 187 (9.6%) patients, and 485 (25%) patients did not have clinical signs of TBI documented in their records. Characteristics of the study cohort are summarized in detail in Table 1.

**Table 1:** Characteristics of the study cohort (*n* = 1941)

Variable		
Age median, years (IQR)	58.7 (39.1–78.5)	
	*n*	%
Men	1122	57.8
Cause of injury		
Ground-level fall	1016	52.3
Motor vehicle accident	261	13.4
Fall from a height	215	11.1
Traffic accident as pedestrian or bicyclist	116	6.0
Other	333	17.2
Alcohol intoxication	567	29.2
Antithrombotic medication	494	25.5
Loss of consciousness	417	21.5
Amnesia	510	26.3
Glasgow Coma Scale		
13–15 points	1100	56.7
9–12 points	97	5.0
3–8 points	81	4.2
Unknown	663	34.2
Traumatic brain injury severity		
No documented clinical signs of traumatic brain injury	485	25.0
Mild	1269	65.4
Moderate to severe	187	9.6
CT positive head injury	368	19.0
Subdural hematoma (acute)	227	11.7
Subdural hematoma (subacute or chronic)	25	1.3
Subarachnoid hemorrhage	216	11.1
Contusion	152	7.8
Cerebral edema	14	0.7
Epidural hematoma	14	0.7
Diffuse axonal injury	7	0.4
CT negative head injury	1573	81.0

*IQR* interquartile range, *CT* computed tomography

Initial head CT showed acute traumatic intracranial pathology and/or any subdural fluid collection (*CT positive*) in 368 (19%) patients. Consequently, 1573 (81%) patients had normal initial head CT in relation to the trauma (*CT negative*)—they had no signs of acute traumatic intracranial pathology or any type of subdural collection on initial head CT.

### CT positive group

In the *CT-positive* group, the most frequent acute traumatic lesion on the head CT was a subdural hematoma, from which 227 (90%) were acute and 25 (10%) subacute or chronic. As subdural hygromas have no unified clinical or radiological criteria, these lesions were included in the subacute or chronic subdural hematoma group. Only 38 (17%) of all acute subdural hematoma (aSDH) patients received acute surgical treatment for the condition. From the 38 patients who received surgical treatment for aSDH, three (7.9%) underwent a future surgery for cSDH. Of the 189 conservatively treated aSDH, nine patients (4.8%) underwent future surgery for cSDH. These findings are presented schematically in Fig. 1.

### CT negative group

The *CT negative* group included 81% of the cohort. The demographics of the *CT negative* group were similar to the characteristics of the entire cohort with the exception that there were markedly fewer patients with moderate to severe TBI in the *CT negative* group. Characteristics of the patients with normal initial head CT are summarized in detail in Table 2.

**Table 2:** Characteristics of the patients with normal initial head computed tomography (*n* = 1573)

Variable		
Age median, years (IQR)	56.2 (36.7–77.2)	
	*n*	%
Men	887	56.4
Cause of injury		
Ground-level fall	804	51.1
Motor vehicle accident	240	15.3
Fall from a height	169	10.7
Traffic accident as pedestrian or bicyclist	84	5.3
Other	276	17.5
Alcohol intoxication	455	28.9
Antithrombotic medication	376	23.9
Loss of consciousness	295	18.8
Amnesia	407	25.9
Glasgow Coma Scale		
13–15 points	938	59.6
9–12 points	55	3.5
3–8 points	24	1.5
Unknown	556	35.3
Traumatic brain injury severity		
No documented clinical signs of traumatic brain injury	483	30.7
Mild	1061	67.5
Moderate to severe	29	1.8
Chronic subdural hematoma within 6 months post-injury	6	0.38
Surgically treated chronic subdural hematoma	2	0.13

*IQR* interquartile range

### Chronic subdural hematoma after normal initial computed tomography

Only six patients were diagnosed with cSDH after a normal initial head CT scan during the six-month follow-up period. Two of the six patients received surgical evacuation of the hematoma (trepanation) while the rest were treated conservatively. Therefore, the incidence of surgically treated cSDH after a normal initial head CT during a six-month follow-up was 0.13% (95% CI 0.023–0.45). Both patients suffered initially from a mild TBI. Therefore, the incidence among patients with mild TBI was 0.19% (95% CI 0.034–0.67). None of the patients with moderate to severe TBI (*n* = 29) or patients with no TBI (*n* = 483) were diagnosed with cSDH during the follow-up period. One of the two patients was on antithrombotic medication (warfarin) at the time of trauma; hence, the incidence among patients on antithrombotic medication (*n* = 376, 24%) was 0.27% (95% CI 0.014–1.5). In addition to these two cases, one patient (a woman, aged 75 years) developed a subdural hygroma which was treated via trepanation shortly after trauma (ten days). Consequently, of the 1573 patients with *CT negative* head injuries, three were surgically treated for subdural fluid collection. Due to the small number of cases that underwent surgery (*n* = 2), statistical analyses on possible risk factors for surgically treated cSDH were not performed.

Both patients who developed surgically treated cSDH were elderly women, aged 77 and 78 years. Hence, the incidence of cSDH in the age group of over 70 years old (*n* = 506) was 0.40% (95% CI 0.070–1.4). Ground-level fall was the injury mechanism for both. Both had bilateral cSDHs. Time intervals from trauma to trepanation for the cSDH were seven and eight weeks. The characteristics of the two patients with surgically treated cSDH are described in detail in Table 3.

**Table 3:** Detailed description of the two patients with surgically treated chronic subdural hematoma after normal initial head computed tomography within six months post-injury (*n* = 2)

Patient	Injury mechanism	TBI severity	Antithrombotic medication at the time of trauma	cSDH trepanation, time since injury	cSDH side	cSDH symptoms
Woman, age 78	GLF	Mild	Warfarin	8 weeks	Bilateral	Postural instability, vertigo, aphasia, fatigue
Woman, age 77	GLF	Mild	No	7 weeks	Bilateral	Postural instability

*GLF* ground-level fall, *TBI* traumatic brain injury, *cSDH* chronic subdural hematoma

## Discussion

### Summary of the key findings

The incidence of surgically treated cSDH after head injuries in adults with normal initial head CT during six-month follow-up was 0.13% in our cohort. The incidence of surgically treated cSDH among patients with antithrombotic medication was 0.27% and 0.40% among patients over 70 years old.

### Comparison of the current findings to prior literature

The prevalence of abnormal initial CT scan after a mild head injury has been reported to range from 6.9 to 29% [15, 18, 21] in the prior literature. In our cohort, 19% had acute intracranial pathology or any type of subdural fluid collection present in the initial CT scan.

The overall incidence of cSDH is well reported in the prior literature. The annual incidence of cSDH in an adult population was 18/100,000 in a study performed in the same region as ours [29]. The incidence was very low in patients under 60 years of age but was significantly higher in older age groups. The incidence was highest in people aged 80 years or older (130/100,000/year), in which it nearly tripled within the study period of 25 years from 1990 to 2015.

Instead, little is known about the development of cSDH in patients with head injuries who have normal initial head CT scans. The only study looking at consecutive CT-scanned patients after head trauma has been published recently by a Japanese group. Karibe et al. examined cSDH formation after mild head trauma in elderly (> 65 years) Japanese patients. Among patients with normal initial CT scans (*n* = 322), surgically treated cSDH was diagnosed in 4.3% of patients at more than one month after injury [19]. In this study, antithrombotic medication did not increase the risk for cSDH although antithrombotic medications were not discontinued or counteracted.

Additionally, a few case reports on this topic have been published. Chia et al. reported a case of an 84-year-old man with a normal initial CT scan after a minor head injury who developed a symptomatic cSDH after two months [5]. The patient was not on antithrombotic medication at the time of injury. Deitch et al. reported on two patients with normal initial CT scans after minor head injury and the development of cSDH after several weeks[8]. The use of antithrombotic medication was not documented. Snoye et al. published a case report of three patients suffering a minor head injury with normal initial head CT scans and the development of cSDH in a mean time of seven weeks[31]. The use of antithrombotic medication was not documented. Kim et al. published a case report of an 82-year-old man with normal initial CT and MRI scans after a mild head injury who was diagnosed with a cSDH after five weeks [20]. The patient was not on antithrombotic medication at the time of injury.

Our study had a notably lower incidence rate of surgically treated cSDH compared to the results published by Karibe et al. The lower incidence rate could be related to the nature of our cohort. The mean age among *CT negative* patients in our study was 56 years, while Karibe et al. reported a considerably higher mean age of 82 years for the whole cohort. The incidence of cSDH has been shown to increase steeply with age [29]. Therefore, the large difference in the mean ages of our samples might partly explain the notable difference in cSDH incidences. In our study, both patients with a surgically treated cSDH were elderly women, aged 77 and 78 years. The incidence of cSDH in the age group of over 70 years old was 0.40%, still considerably lower than the incidence reported by Karibe et al. Another factor that might have affected to the incidences of our studies was the different follow-up practices. Karibe et al. screened all patients with head CT scans one-month post-injury for the detection of cSDH and followed the patients up to 12 months for the detection of symptomatic cSDH, while there was no structural follow-up in our study. Although the main outcome result in our study was the surgical treatment of cSDH, an outcome that should be present with all patients with symptomatic cSDH, some cSDH cases might have been undiagnosed.

The use of antithrombotic medication has been considered one of the primary reasons for the increasing incidence of cSDH in the elderly [29]. The incidence of surgically treated cSDH among patients with antithrombotic medication was 0.27% in all *CT negative* patients. Overall, the risk for delayed intracranial hemorrhage after head trauma seems to be low based on the prior literature. A recent meta-analysis studied delayed intracranial hemorrhage with patients on direct oral anticoagulants (DOAC) (*n* = 1263) and warfarin (*n* = 1788). Delayed intracranial hemorrhage was reported in 2.4% of the patients on DOAC and in 2.3% on warfarin after blunt head trauma [28]. In this meta-analysis, the duration of the follow-up periods varied, with the maximum being just one month. Ghenoweth et al. reported an intracranial hemorrhage incidence of 0.4% for patients without any antithrombotic medication after blunt head trauma in a follow-up period of two weeks [4]*.* These studies examined all intracranial hemorrhages, while our study focused purely on the development of cSDH, and those studies had very short follow-up periods in relation to the development of cSDH. Our study supports the idea that head trauma patients with normal initial head CT do not require routine follow-up CT. The risk for symptomatic cSDH among patients with antithrombotic medication seems to be very low as well.

There remain uncertainties relating to the etiology of cSDH. In 1857, Virchow [23] described the condition as “pachymeningitis hemorrhagica chronica interna”, and at that time, this inflammation theory was widely accepted. Subsequently, a traumatic etiology [25] has been emphasized. Recently aging and brain degeneration [24] have been highlighted as principal causative factors underlying the condition. Although we cannot draw conclusions based on our results about the etiology, the extremely small incidence in our study partly questions the purely traumatic etiology of cSDH.

### Strengths and limitations

To our knowledge, no similar study has been performed previously. Rigorous analysis of the head CT scans was performed to examine only patients with genuinely normal head CT regarding the primary trauma. All the initial head CT scans were evaluated by two neuroradiologists. *CT negative* patients had no signs of acute traumatic intracranial pathology or any type of subdural collection on initial head CT.

Our retrospective patient cohort represents an extensive series of consecutive patients from one geographically well-defined area. All patients were evaluated in the emergency department of one university hospital. Our study was not population-based, though it reflects the incidence of surgically treated cSDH in head trauma patients in Pirkanmaa, Finland. Our cohort was relatively large with 1573 *CT negative* head injuries.

Our study has several limitations. Due to the retrospective design, not all desired data was available. CT scanning practice might have influenced to the composition of our cohort because only CT-scanned patients were recruited to the study. However, our sample included a significant number of patients who were scanned without clear clinical signs of TBI documented in their medical records. The sample likely captures the full severity spectrum of head injuries treated in a university hospital’s emergency department. Additionally, there was no systematic follow-up protocol for the development of cSDH. Most of the cSDH-suspected patients were evaluated by a neurosurgeon and CT scanned only if signs or symptoms of a neurologically treatable condition emerged. Although patients, family members, and health care professionals are informed to contact the neurosurgical department if new worrisome signs or symptoms emerge, some cSDH cases might have been undiagnosed. This is particularly true for patients with only mild or no symptoms. Hence, the main outcome variable was specified as surgically treated cSDH because these patients were most reliably identified. Further, our study was limited by the sample size. The very low number of cases with the outcome of interest increases the uncertainty of the results. Hence, generalization of these results to a larger scale is challenging. Studies with larger patient cohorts are needed to validate the results. Additionally, we were not able to conduct group analyses on possible risk factors for surgically treated cSDH due to the small number of cases. This study did not analyze the development of cSDH among patients with subdural hygromas on the initial CT scans due to the lack of univocal criteria for subdural hygromas. This is an interesting research question and should be specifically assessed in future studies.

### Directions for future research

Similar studies with larger patient cohorts are needed to replicate or validate the results. Epidemiological studies on populations with verified head traumas and case–control matching with patients without trauma could provide interesting information about traumatic vs. non-traumatic etiologies of cSDH.

### Conclusions

The incidence of surgically treated cSDH after head injury in people with normal initial head CTs during a six-month follow-up was minute (0.13%) in our cohort. The extremely low incidence of surgically treated cSDH after a normal initial head CT, even in patients on antithrombotic medication, supports the notion that routine follow-up imaging after an initial normal head CT is not indicated to exclude the development of cSDH. Additionally, our findings support the concept of cSDH not being a purely head trauma-related disease.

## Data Availability

The data that support the findings of this study are available from the corresponding author upon reasonable request.

## Code availability

The code and analyses used are available from the corresponding author upon reasonable request.
